# Reducing Risks to Native Pollinators by Introduced Bees: A Review of Canada’s Legislation with Recommendations for Yukon Territory

**DOI:** 10.3390/biology14030282

**Published:** 2025-03-11

**Authors:** Maria Leung, Donald Reid

**Affiliations:** 1 Wild Tracks Ecological Consulting, Whitehorse, YT Y1A 5T2, Canada; 2 Wildlife Conservation Society Canada, Toronto, ON M5S 3A7, Canada; dreid@wcs.org

**Keywords:** boreal agriculture, native pollinator conservation, bumble bee, foraging lease, honey bee, recommended legislation, pathogens, Canada, Yukon

## Abstract

Imported, non-native honey bees and bumble bees threaten native pollinators by spreading pathogens (disease and parasites) and outcompeting native pollinators for nectar and pollen. We reviewed Canadian federal, provincial/territorial, and municipal legislation to find governance requirements that potentially reduce these threats. We classified the requirements as follows: tracking the number and location of honey bee hives (registry); controlling the spread of pathogens (registry with inspections, quarantines, and cleaning regimes); controlling the competition with native pollinators (limiting shared use of space); and making regulations applicable to all domesticated bees in addition to honey bees. Policies and regulations to control the competition from honey bees are generally lacking. So, we propose the concept of “foraging leases” to manage the location and duration of honey bee apiaries on public lands. Based on the identified requirements, we recommend amendments to the legislation in Yukon Territory, a jurisdiction that still has healthy native pollinator communities that pollinate various human food and medicine crops. Recommendations include tracking hives and their pathogen status with beekeeping regulations enabled by the Animal Health Act, controlling the use of imported bumble bees with changes to the Animal Protection and Control Act and/or the Wildlife Act, and restricting use of public lands for apiaries with the Public Lands Act.

## Simple Summary

Natural ecosystems support a rich variety of native bees, many of which are important economically. Honey bees, which are not native to North America, and domesticated bumble bees spread pathogens (diseases and parasites) and outcompete native bees for nectar and pollen. We aimed to reduce these threats by finding Canadian provincial legislation that could direct legislation in Yukon Territory, where native pollinator communities are still healthy and essential for pollinating agricultural crops. We classified the legislative requirements as follows: tracking numbers and locations of honey bee hives (registry); controlling the spread of pathogens (inspections, quarantines, and cleaning); controlling the competition with native pollinators (limiting shared use of space); and applying regulations to all domesticated bee species. There is little Canadian legislation controlling the interactions of honey bees and native pollinators; the competition problem is poorly addressed. We recommend controls on the numbers, locations, and timing of honey bee hives on public lands in the form of permitted “foraging leases” (for domestic insect livestock), similar to “grazing leases” (for domestic mammalian livestock). We detail changes to Yukon legislation (Animal Health, Animal Protection and Control, Wildlife, and Public Lands Acts) to deal with pathogen spread and competition. Protecting native pollinators will conserve the successful pollination of flowers producing foods and medicines for people.

## 1. Introduction

Pollinators are necessary for the functioning and stability of terrestrial ecosystems, and natural ecosystems function well with native pollinator species [1]. The most efficient pollinators, the bees, are experiencing a disproportionate amount of global insect decline [2]. Two factors contributing to this are (i) novel pathogens transmitted by, and (ii) competition with, honey bees and managed bumble bees.

The honey bee (*Apis mellifera*), a domesticated species not native to North America, can infect native bees and other arthropods with various pathogens (e.g., deformed wing virus, sac brood virus, and small hive beetle) [3,4,5]. Commercially raised bumble bee species (*Bombus* sp.) carrying *Nosema bombi*, a microsporidian parasite, are implicated in the decline of several native bumble bee species [6,7]. Less understood are the impacts of imported alfalfa leaf-cutter bees (*Megachile rotundata*) and blue orchard mason bees (*Osmia lignaria*) [8] that are closely related to native *Megachile* and *Osmia* species in northern Canada.

Honey bees and introduced bumble bees also outcompete and displace native pollinators and disrupt plant–pollinator networks [9,10]. Honey bee hives typically begin with 10,000 worker bees in spring and grow throughout summer. The displacement of native bumble bees by introduced bumble bees has occurred in Canada. The common eastern bumble bee (*Bombus impatiens*), which is used in commercial greenhouses, has expanded its range into southern British Columbia and Washington State [11] and is the most recorded bumble bee in the Greater Vancouver Area of British Columbia [12].

Yukon Territory, Canada, is mostly in the northern boreal biome, with a relatively low human population density, low extent of private agricultural land, and high extent of wild lands under public (i.e., territorial government) jurisdiction compared with many parts of Canada. It is a jurisdiction where native pollinator populations are still largely intact and perform a vital pollination service for human food sources in both wild and agricultural ecosystems, including various berry crops [13,14,15]. Native pollinator species (e.g., *Bombus* spp.) are more cold-tolerant than imported honey bees, and thus, are better adapted to provide this pollination service [15]. Imported honey bees and bumble bees have limited agricultural functions in pollinating field and greenhouse crops and in honey production [16]. Pro-active management of these imported species has the best chance of conserving native pollinators in this context.

Four bumble bee species that occur in Yukon have been assessed by the Committee on the Status of Endangered Wildlife in Canada [17,18,19,20]. The main threats identified for these bumble bees are pathogens, pesticides, climate change, invasive/problematic species, and habitat loss. These threats also pose risks to the native pollinator community in general. In the Recovery Strategies and Management Plans for the four at-risk bumble bees, a high priority is placed on addressing the threats by creating, amending, or influencing environment-related provincial or territorial laws and/or regulations, policies, and guidelines. This legislative approach makes sense for Yukon, which already has some legislation resembling that of the provinces where beekeeping is more tightly controlled to reduce the risk of pathogen spread. Internationally, this legislative approach aligns with the Kunming–Montreal Global Biodiversity Framework’s targets 6, 11, and 14 [21], which aim to mitigate the impacts of alien species, maintain pollination services, and integrate the valued contributions of biodiversity into legislation. It would also help fulfil Canada’s 2030 Nature Strategy that aims to address these targets [22].

Our aim was to review legislation and regulations from federal, provincial/territorial, and municipal governments across Canada for examples of how the threats (pathogen introduction; competition) posed by non-native pollinators (honey bees, domesticated bumble bees) can be reduced and managed so that we can (i) recommend changes to current legislation and regulations in Yukon Territory and (ii) provide a review and general recommendations for improvements to legislation in other Canadian and North American jurisdictions.

We found that the most useful regulatory and management approaches include the mandatory registration and monitoring of honey bee apiaries, prescriptive rather than permissive approaches to commercial extractive uses of public lands, the concept of foraging leases for honey bees on public lands, and seasonal limitations on the use of domesticated bumble bees in greenhouses.

## 2. Materials and Methods

To understand how Canadian governments currently aim to reduce the threat of pathogen spillover to, and competition with, native pollinators, we examined the legislation governing beekeeping and the use of other managed bees at the federal and provincial/territorial levels.

We searched Canadian federal legislation for regulations dealing with bees, which led to the work of the Canadian Food Inspection Agency (CFIA) under the Health of Animals Act. The 10 provinces and 3 territories in Canada have jurisdiction over most animals and lands, so we searched their legislation for the governance of honey bees, other domesticated and imported bees, animal pathogens (particularly for honey bees), and beekeeping on public lands. This involved searches through the consolidated statutes for each jurisdiction.

The “legislation” consists of acts, regulations, and enabling statutes supplemented by other legally enforceable policies, such as protocols. This legislation can be implemented by the federal, provincial/territorial, or municipal levels of government (see the Results Section), though the bulk of it is provincial (Table 1).

Though we recognize that the main purpose of honey bee legislation and regulation is to protect the health of honey bees and the honey bee industry (including by reducing the risks of pathogen transmission between honey bee hives), many of these precautions also reduce the risk to wild pollinators by averting the introduction and spread of diseases and pests that potentially affect wild pollinators.

We categorized the legislative requirements by general function or intent (termed “requirement”) with respect to their benefit to native pollinators. We considered five categories: tracking the number and location of honey bee hives; controlling the spread of pathogens; controlling the competition with native pollinators; reducing the lethal and sub-lethal effects of pesticides; and making regulations applicable to domesticated bees other than honey bees. Within each category of requirement, we itemized the various actions required to satisfy the intent. For each requirement and action, we documented the example wording and noted additional clauses that could potentially abate threats. No dedicated legislation exists for beekeeping in Yukon, but what other legislation does exist was examined for its applicability to the categories identified from the provincial pieces of legislation.

We also tabulated the pathogens identified by each province and whether they were considered reportable in each jurisdiction. The intent here was to understand the scope and sufficiency of the listing processes.

We then identified the requirements within municipal bylaws. Many municipalities have bylaws regulating beekeeping in urban areas. These are often less prominent than provincial/territorial governance but can still potentially reduce the risk of pathogen spillover to, and competition with, native pollinators. An exhaustive search of all municipalities across Canada was beyond the scope of this study. Instead, municipal bylaws and regulations were collected for the northern territories where available, along with a selection of representative ones from each province, if available.

To understand what legislation exists to reduce the risks associated with introduced bumble bees competing with, or spreading pathogens to, native bumble bees, we identified provincial and territorial legislation that complements the federal Species at Risk Act (SARA) and examined it for its relevance to pollinator species at risk. Other federal legislation relevant to the use of managed bumble bees was also identified, which was mainly legislation related to the importation of live organisms into Canada or into individual provinces.

## 3. Results

Three levels of government—federal, provincial/territorial, and municipal—have legislation relevant to honey bees, managed bumble bees, or other introduced bees. The federal government is responsible for regulating the import of organisms into Canada and the reporting of pathogens listed by the World Organisation for Animal Health. It is also responsible for wildlife species at risk on federal lands. The provinces and territories have the most jurisdiction over domestic livestock, such as honey bees, and animal husbandry, such as beekeeping. They also have jurisdiction over what may enter the province or territory, and over management of non-federal public lands and species at risk on non-federal lands. Municipalities govern activities within urban boundaries, including activities that have traditionally been carried out on rural lands, such as the raising of chickens or pigs and beekeeping.

### 3.1. Federal

For all of Canada, the federal Health of Animals Regulation (Consolidated Regulations of Canada, c. 296), enabled by the Health of Animals Act (Statutes of Canada, 1990, c.21), applies. The Canadian Food and Inspection Agency (CFIA) enforces the regulations and issues import permits for honey bees that meet the requirements of permissible source countries and any other measures intended to reduce the spread of pathogens [23]. Applications to import other species of bees, such as blue orchard mason bees and bumble bees, into Canada are assessed on a case-by-case basis under the authority of the Plant Protection Act (Statute of Canada, 1990, c.22) and CFIA Directive 12-02 [24]. The CFIA prohibits importation of alfalfa leaf-cutting bees from outside Canada due to the risk of pathogens, including chalkbrood [25]. Only one provincial region, Newfoundland and Labrador, also prohibits the importation of alfalfa leaf-cutting bees (which could come from other provinces), as well as all other managed bees (e.g., bumble bees, blue orchard mason bees) aside from honey bees; this is regulated by the Wild Life Regulations (Consolidated Newfoundland and Labrador Regulation 1156/96 s.83).

### 3.2. Species at Risk Legislation

Species at risk legislation governs the assessment process for identifying wildlife species at risk and implementing conservation actions. Federally identified wildlife species at risk do include some native pollinators, such as the Gypsy Cuckoo Bumble Bee (*Bombus bohemicus*) and McKay’s Bumble Bee (*Bombus mckayi*). However, Canada’s federal Species at Risk Act only applies to federal lands, such as National Parks. In Yukon, there is no complementary territorial Species at Risk Act for territorial public lands, which account for approximately 85% of the Yukon’s land base [26]. Six provinces and one territory have some form of a species at risk act (Table 2) [27]. All the provinces with species at risk legislation have included at least some insect pollinators in their lists of species at risk. These pollinators include bees, butterflies, and moths. Northwest Territories’ Species at Risk Act has enabled the assessments of three bumble bees that are listed federally and in some other provinces but has not included them in the resulting list of species at risk (Table 2).

### 3.3. Provincial and Territorial Legislation

All provinces have legislation that actively governs beekeeping. Six provinces have statutes specific to bees or apiaries (apiary: a collection of honey bee hives). The other four provinces—British Columbia, Quebec, Newfoundland, and Prince Edward Island—use their statutes on animal health to enable regulations pertaining to beekeeping (Table 1). Additional statutes also support beekeeping in some provinces. For example, despite Saskatchewan having an Apiaries Act, its Animal Health Act and Regulations control the reporting of bee pathogens. None of the three territories—Yukon, Northwest Territories, or Nunavut—has territorial statutes specific to beekeeping or beekeeping regulations. However, the Yukon’s Animal Health Regulation (O.I.C. 2018/159) lists mandatory reporting of suspected or confirmed cases of two honey bee “transmissible hazards”: American foulbrood (*Paenibacillus* larvae) and the small hive beetle (*Aethina tumida*).

From the statutes and regulations overseeing beekeeping, we identified sets of requirements that are of benefit to native pollinators (Table 3) while facilitating the management of the honey bee industry. These legislative requirements, and associated actions, comprise a systematic assessment of governance functions.

The first requirement is the obligation to track the number and location of honey bee hives, including the following specific actions: register hives; identify individual hives; register the specific location of each hive; and report all movements of hives or colonies (Table 3). Except for Prince Edward Island, all provinces require beekeepers to register their hives. Information collected during registration usually includes the number and location of hives, which is essential in tracking and containing pathogens, and may provide a density estimate of honey bees on the landscape. For the adequate tracking and containment of the threats of pathogen spread and competition, we consider all four of these actions to be necessary.

The second requirement is to control the spread of diseases and pests (pathogens). This category includes various legislated actions, not all of which are mandatory or enacted in every province. These actions include restrictions on the sources of honey bees, quarantines, inspections of colonies, sanitization of equipment, and mandatory reporting of diseases and pests. All provinces control the importation of honey bees and beekeeping equipment from other provinces, and in the case of British Columbia, the movement of hives within the province is closely regulated, allowing for quarantine zones when pathogen outbreaks occur.

The third requirement, to control competition between honey bees and native pollinators, has had very little legislative attention (Table 3). British Columbia is the only province to have specific regulations for apiaries on public (“Crown”) land. These allow the province to control the locations, densities, and duration of privately owned hives on public lands, all of which are necessary tools to influence the competition between honey bees and the already established native pollinator community. This approach is easier with public lands legislation that is prescriptive (i.e., prohibiting any private use of public lands unless that use is explicitly allowed with conditions detailed in the legislation) as compared with permissive (i.e., allowing all private uses of public land with controls and prohibitions only occurring when specifically addressed in the legislation). Restrictions on the number of hives on any piece of land were generally absent in provincial legislation, but common at the municipal level, which is discussed later.

The fourth requirement, to reduce the negative effects of pesticides on bees, is generally addressed in legislation other than what we reviewed and was beyond the scope of this study. However, it is worth mentioning that Ontario’s Bee Act and Quebec’s Animal Health Protection Act both prohibit the spraying of fruit trees with any substance toxic to bees while the trees are in bloom.

The fifth requirement is to make regulations applicable to all domesticated bee species, including honey bees (Table 3). This has had relatively little legislative attention, partly because importation into Canada is controlled by federal legislation (see above). Some provinces list various domesticated species, as well as honey bees, giving the government the option to act on threats these domesticated species pose.

Returning to the question of pathogen reporting and control (the second requirement), all provinces have identified some pathogens associated with beekeeping. These pathogens are listed in provincial legislation used to facilitate their control and eradication. The pathogens, including disease organisms and pest species, listed from provinces across Canada comprise the following numbers of species: five mites, five honey bees, four wasps or hornets, three beetles, two wax moths, one louse, three fungi, two bacteria, and eight types of virus (Table 4). All 10 provinces list the small hive beetle, Varroa mite, and American foulbrood. Only British Columbia and Manitoba identify viruses in their legislation. Some provinces specifically list “reportable” and “notifiable” pathogens. “Reportable” pathogens require immediate action to contain their spread. “Notifiable” pathogens are monitored to help understand their presence and impacts. In provinces where notifiable and reportable pathogens are named, the beekeeper is obligated to report suspected cases within a limited time.

At 23, British Columbia identified the most pathogens in its legislation, followed by Alberta and Ontario at 18. All provinces have at least one reportable or notifiable pathogen, with the exception of Alberta, which has no honey bee pathogens listed in its Reportable and Notifiable Diseases Regulation (Alberta Regulation 129/2014). The reporting of some pathogens is still recommended in Alberta [28]. The Canadian Federal Inspection Agency also monitors tracheal mites, the small hive beetle, American foulbrood, and European foulbrood (Health of Animals Regulations, Consolidated Regulations of Canada, 2024, c. 296). Mandatory reporting by laboratories across Canada contributes to this monitoring effort.

### 3.4. Municipal Legislation

Municipal legislation pertaining to beekeeping is generally more developed in western Canada than in central or eastern Canada, with British Columbia having upward of 20 municipalities permitting and regulating beekeeping. There were considerable similarities between bylaws, and the sample (see Table S2 in the Supplementary Materials) appeared sufficient to identify the main requirements. Regulations are usually enabled by animal control bylaws (e.g., Edmonton Animal Licensing and Control Bylaw No. 13145), zoning bylaws (e.g., Winnipeg Zoning Bylaw), or dedicated urban beekeeping bylaws (e.g., Chilliwack Urban Beekeeping Bylaw 2019, No. 4680).

The focus of the regulations is on keeping the public safe from the defensive behavior of honey bees, especially bee stings. Regulations usually stipulate the maximum number of beehives for the size of the property and the placement of beehives relative to property boundaries, and often mention having the hive entrance directed away from adjacent properties and/or requirements for a physical barrier, such as a fence or hedge to discourage the direct flight of honey bees to adjacent properties (e.g., Municipal Planning Guide to Zoning Bylaws in Manitoba [29]). A few municipalities require proof that the applicant has taken a beekeeping course or is being mentored by an experienced beekeeper (e.g., Mount Pearl Development Regulations 2010).

The two municipalities in Yukon that explicitly allow beekeeping use different bylaws to regulate the activity. The City of Whitehorse uses its Zoning Bylaw 2012–20 to specify where, within the city limits, beekeeping is permitted. Beyond this, there are no published regulations on the placement of beehives within properties or the permissible number of beehives and no permitting process to keep track of beekeeping activity. The beekeeping regulations in Dawson City are enabled by its Animal Control Bylaw and are similar to the municipalities in southern Canada, with well-developed beekeeping regulations specifying the maximum number of beehives, placement, and management practices to reduce risks to neighbouring properties. Dawson City has a permitting process in place that provides a record of the beekeeper, where hives are located, and from where they were moved.

## 4. Discussion

### 4.1. The Yukon Context

Having a diversity of pollinators for the large diversity of plants native to Yukon creates a complex plant–pollinator network. The production of northern wild foods, such as blueberries, lingonberries (*Vaccinium* spp.), and cloudberries (*Rubus chamaemorus*), as well as some domestic crops (e.g., haskap (*Lonicera caerulea*), beans (*Phaseolus* spp.), and squash (*Cucurbita* spp.)), depends on these pollinators [13,14,15]. In Yukon, there are over a hundred species of bees; over a hundred species of butterflies; and many more moths, flies, and other pollinator insects [30]. This native pollinator network is thought to be relatively intact compared with networks farther south in Canada and North America because Yukon has a relatively low human footprint. The bumble bees listed in Canada’s Species at Risk Act are believed to be closer to historic levels in Yukon than in other parts of Canada because less of the landscape has been subjected to alteration by agriculture, resource extraction, or other uses. So, now is an appropriate time to act to strengthen the conservation of native pollinator species in Yukon before very many become threatened.

The Canadian legislative framework for reducing threats to native pollinators has improved in recent years. A 2007 review of federal and provincial legislation [31] concluded that Canadian federal and provincial legislation lacked explicit provisions for pollinator conservation, but did have provisions related to pollinator conservation. Since that study, there have been numerous amendments to existing legislation and the introduction of new legislation. Although provisions specific to the conservation of native pollinators are still generally lacking across Canada, there are many examples of legislation that is potentially useful in reducing the risks to native pollinators. In the context of legislation across the country, we examined current Yukon legislation to evaluate its ability to address the threat of pathogen spillover to native pollinators from introduced bees and the threat of competition with native pollinators by introduced bees.

### 4.2. Pathogen Spillover

The main sources of pathogens affecting native bees are imported honey bees, a species not native to Canada, and commercially raised and imported bumble bees (mainly *Bombus impatiens*) used in greenhouse operations [32,33]. Pathogens and parasites weaken native pollinators, and when pervasive, or combined with other stressors, are capable of reducing the populations of native pollinators [1]. The pathogens associated with honey bees have been found in arthropods other than bees, likely transmitted at floral feeding sites or by predation on infected honey bees [3].

Compared with the provinces, Yukon Territory has very little legislative infrastructure to protect native pollinators from pathogen spillover imposed by honey bees and other managed bees. This makes Yukon vulnerable to the importation of infected honey bees from other Canadian jurisdictions. The territorial legislation is limited to the mandatory reporting of two “transmissible hazards”: American foulbrood and the small hive beetle (Table 4). Dawson City is the only region in Yukon that reinforces this mandatory reporting.

Mandatory reporting is difficult to enforce without a system to register beekeepers and the location of their hives and a system to inspect and track imported honey bees. Unlike all the provinces, Yukon does not monitor honey bees or beekeeping equipment being moved into the territory and does not systematically regulate from where honey bees may be imported. Though, like the rest of Canada, Yukon beekeepers should be complying with the federal Health of Animals Regulation (C.R.C., c. 296), which includes restrictions on which countries honey bees can be imported from. For many years, a long-time beekeeper in Yukon was a proponent of sourcing honey bees from mite-free colonies to keep Yukon free of varroa mites [16], but there was no legislation to support this initiative. Today, honey bees in Yukon are commonly infected by varroa mite [16] and this could be widespread throughout Yukon. Beekeeping is widespread in Yukon, ranging north to Dawson City, west to Haines Junction, east to Watson Lake, and south to Teslin [34]. However, the actual number of beekeepers and number of hives remains unknown because the territory lacks a formal registry.

The pathogens associated with honey bees that could enter Yukon are not reflected in the territory’s current legislation. The list of two pathogens identified for honey bees by Yukon’s Animal Health Act (Statutes of Yukon 2013, c.10) is shorter than any province, and notably far less than the bordering provinces of British Columbia, with 23 listed pathogens, and Alberta with 18 (Table 4). It could be argued that the mandatory reporting of select pathogens in British Columbia offers some protection to Yukon; mandatory reporting in British Columbia facilitates the isolation and disinfection of affected hives. The same cannot be said of Alberta, where reporting is recommended but not legislated [28].

Across Canada, legislation abating the risk of pathogen spillover from managed bumble bees is less developed than that from honey bees. The necessary management approaches to mitigate the risks of managed bumble bees to native bees are as follows: the monitoring of pathogens in commercial bumble bee stock, preventing escape from greenhouses, and regulating the movement of commercial bumble bees [33]. Newfoundland’s prohibition of any species other than those listed in their Wild Life Regulations (Consolidated Newfoundland and Labrador Regulation 1156/96 s.83) is the strictest provision pertaining to the movement of managed bumble bees. It prohibits any importation of bumble bees and is, in part, enforceable because it defines “wild animal” as “any live animal, including without limitation, any amphibian, arthropod, bird, coelenterate, crustacean, fish, other invertebrate, mammal, mollusk or reptile, whether or not bred, hatched or born in captivity and including any egg or offspring of them”. By contrast, the Animal Protection and Control Act of Yukon (Statutes of Yukon, 2022, c.13) currently does not enable the control of any invertebrates. Furthermore, the Wildlife Act (Revised Statutes of Yukon 2002, C.229) for Yukon only includes vertebrate species in its definition of “wildlife”, so is unable to offer any protection to invertebrates, such as insect pollinators.

Another avenue for protecting native pollinators from pathogen spillover is possible, specifically for pollinator species listed in Canada’s Species at Risk Act (SARA). When species are listed under the SARA, the federal government is obligated to prepare Recovery Strategies for “endangered” and “threatened” species, and Management Plans for “special concern” species. Such action plans identify approaches to reducing threats to the species, and it is mandatory to track the progress of these strategies and plans (Species at Risk Act, Statutes of Canada 2002, c.29). An example of an action within a Recovery Strategy to protect a native pollinator against pathogen spillover is the promotion of the Bumblebee Sector Guide to the National Bee Farm-level Biosecurity Standard [35]. This standard provides details on how to confine managed bumble bees to greenhouses and to prevent the spread of pathogens [35].

Action plans enabled by the federal Species at Risk Act (SARA) are limited in their ability to achieve the goal of arresting or reversing species decline. One main limitation is the fact that for terrestrial species, the SARA only applies to federal lands (those “that belong to His Majesty in right of Canada”). In the provinces, this is only 4% of the land [27]. The vast majority of land in Yukon is public land, still vested in the federal Crown but administered under territorial jurisdiction (Yukon Act S.C. 2002, c. 7) [26]. In practice, the application of the SARA in Yukon has come to refer only to federally controlled or administered lands, as is conducted in the provinces. Federally controlled lands in Yukon (mostly conservation lands) comprise c. 8% of the land base [36]. Unlike the Northwest Territories and seven provinces, Yukon does not have legislation that complements the federal SARA (Table 2) [27]. Provincial and territorial equivalents of the federal SARA usually adopt components of federal Recovery Strategies and Management Plans for use on public lands under provincial and territorial administration.

### 4.3. Competition

Unlike native bees, worker honey bees can recruit each other to harvest the best sources of nectar and pollen, thereby monopolizing the food source [10,37]. The level of numerical dominance by honey bees increases with flower abundance [38]. The amount of pollen a honey bee colony collects from June to August is equivalent to the amount needed to produce 100,000 progeny of an average solitary bee [39]. Under such competition from honey bees, the most affected native species would have difficulty maintaining the local population levels into the future.

Legislation that could reduce the risk of resource competition from honey bees and other managed bees is limited, with most examples coming from municipal bylaws. Urban beekeeping is a growing trend in Canada, with many municipalities moving away from the prohibition of beekeeping, not enforcing the prohibition, or developing regulations around beekeeping. Municipal regulations have the potential to control the amount of competition with native pollinators and the risk of pathogen spillover, by either prohibiting beekeeping or by limiting the number of honey bee hives. However, because public safety is the main rationale for restricting the number of bee hives within municipal boundaries, the overall density of bee hives and their risk to native pollinators has yet to be factored in. The risk of competition was highlighted by the finding that the high honey bee density in Montreal was negatively impacting wild bee species richness [40]. Similar findings have been documented from elsewhere in Canada and Europe [41,42]. Beyond municipal boundaries, most provinces track the location and number of honey bee hives by using a registry system, where such registries have the potential to track the density of bee hives and competition risk to native pollinators.

Legislation specifically restricting the placement of apiaries on public lands appears lacking for most provinces. The lack of regulation would indicate it is a prohibited activity if the particular provincial Lands Act is prescriptive in nature, meaning that by default, activities are prohibited unless authorized. British Columbia appears to be the only province that has explicitly authorized regulations pertaining to apiaries on public lands, and these were enabled by its provincial Lands Act (C.245, R.S.B.C., 1996, s.60.). Unlike British Columbia, which has a prescriptive Lands Act, the existing Yukon Lands Acts (Territorial Lands (Yukon) Act, Revised Statutes of Canada, 1985, c. T-7; Lands Act Revised Statutes of Yukon 2002, c. 132) are permissive, allowing any activities to occur without restrictions unless those activities are specifically addressed in the Act or by its enabled legislation. At present, this means that beekeeping on public lands administered by the Yukon Government is permitted without restriction. This may change as the Yukon’s Lands Acts are undergoing major changes [26].

Providing sufficient forage is a general concern and recommendation for beekeepers [43]. Honey bees are essentially free-ranging livestock, travelling beyond property lines to collect nectar and pollen. Unrestricted access to public lands by beekeepers with apiaries potentially creates problems with competition between beekeepers and their honey bees, and competition between honey bees and native pollinators. For other livestock in Yukon, such as cattle, sheep, and horses, competition with wildlife is a consideration in deciding whether a land parcel can be a grazing lease. Adopting the approach of the Yukon Grazing Policy [44] for identifying public lands for grazing livestock could be a starting point for developing an assessment of where suitable sites would be for apiaries on public lands. Specifically, the Yukon Grazing Policy stipulates a minimum amount of available forage within a defined area, and how the period of time that the land can be used for grazing livestock is determined. The policy also summarizes a process to identify conflicting land uses and states that applications for grazing are subjected to review by the Yukon Government or the Yukon Environmental and Socio-economic Assessment Act.

We recommend the establishment of foraging leases as a necessary condition for private honey bee keepers to place apiaries on public lands. By using an approach like the Yukon Grazing Policy, regulators would limit the number of hives to the capacity of the forage (nectar and pollen) in defined areas (foraging leases), and such leases would be limited in time and space. These limitations could help mitigate the competition with native insects reliant on similar nectar and pollen sources.

Management practices need to be in place to reduce the number of apiaries on public lands during time periods outside the period of mass flowering [32]. Mass flowering refers to events during which a set of plant species produce many blooms within a short period of time. Competition with native pollinators would be the most intense before and after such mass-flowering periods when fewer food sources are available [32]. The prohibition of apiaries in protected areas is also recommended to avoid disruption to native pollinators [32]. This is supported by a study that found the carrying capacity of floral resources in protected areas was exceeded by the introduction of honey bees [45]. As is conducted in British Columbia, restricting the window of time during which apiaries can be on public lands and regulating cleanup would help ensure that the public lands will return to their original state as much as possible.

Precedents for limiting the density of honey bees and assessing risk to native pollinators on public lands are generally lacking in Canada. However, there are initiatives in the United States that demand environmental assessments prior to deciding whether to issue permits for apiaries on public land. Such assessments would consider the risk of displacing native pollinators from their essential food sources, and also the risk of potential pathogen spillover from honey bees to native bees [46,47].

### 4.4. Recommendations for Legislative Change in Yukon

Through a combination of amending the existing Yukon legislation and introducing new legislation, it would be possible to reduce the risk of pathogen spillover to native pollinators and mitigate the competition between introduced bees and native pollinators.

#### 4.4.1. Pathogen Spillover

For Yukon, existing legislation can be used to reduce the risk of pathogen spillover from honey bees. The Animal Health Act has the ability to create regulations to prevent the entry and spread of disease to domestic and wild animals. We recommend creating a set of regulations enabled by the Animal Health Act specific to beekeeping. In the regulations, we suggest the inclusion of the following stipulations:

- Registration of apiaries, including the beekeeper’s name, number of hives, and specific location(s) of hives;
- Onsite identification of apiaries to facilitate contact with the beekeeper if needed;
- Reporting of any movement of apiaries and equipment so as to track the potential spread of pathogens;
- Inspection of honey bees and equipment prior to import into Yukon, and certification to ensure that the honey bees and equipment are free of pathogens;
- Updated list of pathogens to include all pathogens at risk to honey bees in the provinces from which the bees are being imported;
- Continued mandatory reporting of “transmissible diseases” as in the current Animal Health Regulations.

To reduce the risk of pathogen spillover from managed bumble bees, there are two options: one that would prohibit the use of managed bumble bees altogether, and another that would control the use of managed bumble bees. We prefer the higher effectiveness of the first option but discuss how to implement either through legislative changes to existing Yukon Acts or Regulations.

Under option 1, prohibiting the use of managed bumble bees in Yukon would either need amendments to the Animals and Species Regulation (Ministerial Order 2024/12) or amendments to Yukon’s Wildlife Act.

Currently, the Animals and Species Regulation enabled by the Animal Protection and Control Act prohibits “an animal that is not livestock and does not belong to an allowed species or a restricted species” (s 6(a)). This wording offers the possibility of preventing the import of non-native bumble bees if the definition of “animal” in the Animal Protection and Control Act were to include non-native bumble bees. That definition currently only includes vertebrates and “prescribed” species, and the “prescribed species” in the Animals and Species Regulation are limited to cephalopods, tarantulas, and scorpions. Adding bumble bees as “prescribed” species to Schedule 1 of the Animals and Species Regulation would enact a prohibition on the import of non-native bumble bees.

For the Wildlife Act to prohibit managed bumble bees, the definition of “wildlife” would need better clarity to include native insects so that stipulations to protect wildlife through the Wildlife Act are applicable to native pollinators. The current definition of “wildlife” as “wild by nature” may or may not apply to native insects. Then, a new section would be needed in the Act to prohibit the importation of any species unless authorized, akin to Newfoundland and Labrador’s Wild Life Regulations (Consolidated Newfoundland and Labrador Regulation 1156/96 s.83). This would enable the prohibition of insects considered a risk to Yukon’s wildlife and insects that are considered invasive.

Under option 2, controlling the use of managed bumble bees to minimize pathogen spillover to native pollinators would require amendments to the Animal Protection and Control Act and its regulations. For the purposes of the Animal Protection and Control Act and the Animal Protection and Control Regulations (O.I.C. 2024/62), livestock is defined by the species in Schedule 2 of the Animals Species Regulation and Schedule A of the Game Farm Regulations (O.I.C. 1995/015). Adding non-native bumble bees as livestock to Schedule 2 of the Animals and Species Regulation would enable the Animal Protection and Control Act to prescribe conditions for the use of managed bumble bees in Yukon. The conditions should include assurances that the bees are only permitted for use in winter (November to March) and that they are confined to greenhouses. This would prevent contact between the managed bumble bees and native bees; native bees are dormant in winter. We also recommend the destruction of the managed bumble bee colonies at the end of winter to prevent any individuals from escaping out of the greenhouses. If the option of a standardized disease-free stock of bumble bees becomes available [48], we recommend the mandatory sourcing of commercial bumble bees from facilities that produce such stock.

#### 4.4.2. Competition

The changes to legislation that we recommend in the Pathogen Spillover Section (Section 4.4.1) are applicable to mitigating the competition between managed bees and native pollinators. Regarding honey bees, the establishment of a registry that can track the location and density of apiaries throughout Yukon is not only useful for understanding where the competition risk to native pollinators is the highest, but also useful for beekeepers wanting to ensure adequate food for their honey bees. Regarding managed bumble bees, by prohibiting the use of managed bumble bees or confining their use to greenhouses in winter, competition with native pollinators would be avoided.

Beyond the recommended changes discussed in the Pathogen Spillover Section, there are additional amendments to Yukon’s territorial legislation and municipal bylaws that could reduce the competition with native pollinators by honey bees. Apiaries are kept on both public and private lands in Yukon, though in both circumstances, the honey bees have access to food on public lands and compete with native pollinators. This competition cannot be avoided altogether while beekeeping continues in Yukon because, unlike livestock that can be confined by fences, honey bees are free-ranging. However, restrictions on the number of hives and the placement of apiaries can reduce the competition with native pollinators from honey bees.

At the municipal level, having municipalities, such as Whitehorse, adopt wording similar to the Dawson City beekeeping regulations into its Zoning Bylaw or Animal Control Bylaw would potentially limit the placement and density of honey bees within the municipality. The Dawson City regulations include a maximum of two hives for properties smaller than 0.41 ha, and a maximum of four hives for properties 0.41 ha or larger. Additionally, imposing a total quota of apiaries within city limits could reduce competition with native pollinators.

At the territorial level, the new Public Lands Act that the Yukon Government is now developing could direct the conditions by which apiaries would be permitted on public lands. At one extreme, apiaries on public lands could be prohibited altogether, and this would produce the least risk to native pollinators. At the other extreme, in what currently exists, apiaries would be permitted without restriction because, by default, activities are allowed unless prohibited by the current Lands Acts and enabled regulations. Somewhere between these extremes would require a regulated approach that mitigates the risk of competition with native species while still allowing some use of public lands. We recommend that the new Public Lands Act be prescriptive, rather than permissive, so that it includes clause(s) that prohibit apiaries on public land except under specific conditions and through a permitting process similar to how grazing agreements are devised in Yukon.

To mitigate the negative consequences of competition with native species, the permitting process would include the following considerations:

- Forage: Identify sites that have a high abundance of floral resources, such as recent burns in forested lands, or repeatedly disturbed sites (rights of way and gravel pits). Estimate the longevity of the abundance, as some sites will lose their abundant floral resources as part of forest succession or changes to land use.
- Timing: Restrict the window of time during which honey bees can be at a site. This could reduce competition against native pollinators when food is most limiting (e.g., early spring), and permit apiaries when flowers are most abundant, and thus, least likely to be limiting to native pollinators.
- Quota: limit the total number of apiaries so that the total competition with native pollinators is reduced.
- Placement A: Avoid remote sites to reduce the chances of pathogen spillover to native pollinator populations that are currently less affected by introduced pathogens and to maintain native plant–pollinator networks without intrusion. Instead, only use sites with easy road access. For native species, road access often indicates that the area is already disturbed compared with a remote site. For beekeepers, road access to sites facilitates the easier placement of hives and removal at the end of the growing season.
- Placement B: Prohibit apiaries in rare ecosystems and protected areas. Disrupting the native plant–pollinator network in ecosystems that are already spatially limited compromises their future. In particular, native grassland ecosystems in Yukon often support rare and/or range-restricted plant species [49], and their pollinator networks are incompletely understood and may contain specialist species.

#### 4.4.3. Species at Risk

Creating species at risk legislation in Yukon would help to implement Management Plans and Recovery Strategies for safeguarding our native pollinators that are federally listed species at risk. Yukon has previously signed on to the 1996 Accord for the Protection of Species at Risk, and the 2018 Pan Canadian Approach to Transforming Species at Risk Conservation in Canada. The Canada–Yukon Nature Agreement [50] states that its purpose “is to establish the framework for cooperation between the Participants for measures and plans by the Participants for the protection, conservation and recovery of biodiversity, habitat, and species at risk in the Yukon”. These initiatives all point to the need and existing mandate for Yukon to enact species-at-risk legislation.

## 5. Conclusions

Given that imported non-native honey bees and bumble bees can threaten the viability of native bee pollinator species, and the native bees’ service of pollinating northern boreal food crops, our review of the Canadian governance regime indicates that legislation needs to (i) track the number and location of honey bee hives; (ii) control the spread of pathogens; (iii) control competition with native pollinators; and (iv) make regulations applicable to all domesticated bees, in addition to honey bees. These four requirements or functions of governance need to occur in provincial or territorial legislation; currently, they are very unevenly satisfied across these jurisdictions within Canada. In particular, policies and regulations aimed at controlling honey bee competition with native pollinators are generally lacking. In response, we propose the concept of “foraging leases” to manage any proposed location of honey bee (i.e., domestic insect livestock) apiaries on public lands because these lands often support a full complement of native pollinators. Foraging leases would be modelled on “grazing leases”, a management tool already well established for the management of domestic mammalian livestock on public lands.

Specific to Yukon Territory, we recommend new regulations under the Animal Health Act to satisfy the need to track the number and location of honey bee hives, and to control the potential spread of pathogens into and within the territory. We explain alternative options for managing domestic bumble bees under the Animal Protection and Control Act or the Wildlife Act, each of which would require the explicit listing of domestic bumble bees as species requiring management. To control the competition for floral resources between introduced bees and native bees, we recommend a new Public Lands Act (currently under development) that is prescriptive and controls all placement of apiaries on public lands within a set of conditions defined by the abundance of forage (flowers), the timing window within the growing season, a quota (density of hives), and the placement with respect to permitted and non-permitted land covers. The resulting spaces and times when apiaries are allowed on public lands could be called foraging leases. We also recommend the establishment of a Yukon Territory Species at Risk Act to provide habitat protection for pollinators already at risk.

## Figures and Tables

**Table 1:** The statutes and regulations governing beekeeping in each Canadian province.

Province	Statute	Regulations
British Columbia	Animal Health Act, Statutes of British Columbia (2014 c. 16) Lands Act, Revised Statutes of British Columbia (1996, c. 245)	Bee Regulation, British Columbia Regulation 3/2015 Reportable and Notifiable Disease Regulation 7/2015 O.C. 14/2025 British Columbia Land Use Policy Permission, 2023
Alberta	Bee Act, Revised Statutes of Alberta (2000, c. B-2)	Bee Regulation, Alberta Regulation 194/2003
Saskatchewan	The Apiaries Act, Statutes of Saskatchewan (2005, c. A-22.01) The Animal Health Act, Statutes of Saskatchewan (2019, c. A-20.01)	The Apiaries Regulations, Saskatchewan Regulations, 2005 The Animal Health Regulations, 2019
Manitoba	The Bee Act, Continuing Consolidation of the Statutes of Manitoba (1988, c. B15) Animal Diseases Act, Continuing Consolidation of the Statutes of Manitoba (2022, c. A85)	Diseases of Bees Designation Regulation, 2024 Reportable Diseases Regulation, 2007
Ontario	Bees Act, Revised Statutes of Ontario (1990, c. B-6)	Regulation 57 General, Revised Regulations of Ontario 1990
Quebec	Animal Health Protection Act, Revised Statutes of Quebec (1964, P-42)	Regulations Respecting the Registration of Beekeepers, 2024 Regulation Respecting the Inscription Affixed on Hives, 2024 Regulations to Designate Contagious or Parasitic Diseases, Infectious Agents and Syndromes, 2020
New Brunswick	Bee Act, Statute of New Brunswick (2021, c. 21)	New Brunswick Regulation 13/2023
Nova Scotia	Bee Industry Act, Statutes of Nova Scotia (2005, c. 3)	Bee Industry Regulations, Nova Scotia Regulation 133/2012
Prince Edward Island	Animal Health Act, Revised Statutes of Prince Edward Island (1988, c. A-11.1) ^1^	Bee Health Regulations, 2019
Newfoundland	Animal Health and Protection Act, Statutes of Newfoundland and Labrador (2013, c. 1)	Animal Health Regulations, 2012 Animal Reportable Diseases Regulations, 2012

^1^ This is under revision (An Act to Amend the Animal Health Act Bill No. 45, 2024).

**Table 2:** Provincial and territorial statutes for species at risk and a list of any pollinator species to which the legislation applies.

Province	Statute	Listed Pollinator Species
British Columbia	None	
Alberta	None	
Saskatchewan	None	
Manitoba	The Endangered Species and Ecosystems Act, Continuing Consolidation of the Statutes of Manitoba (1989–1990, c. 39)	Dakota skipper (*Hesperia dacotae*) Ottoe skipper (*Hesperia ottoe*) Uncas skipper (*Hesperia uncas*) Dusky dune moth (*Copablepharon longipenne*) Pale yellow dune moth (*Copablepharon grandis*) Poweshiek skipperling (*Oarisma poweshiek*) Verna’s flower moth (*Schinia verna*) White flower moth (*Schinia bimatris*)
Ontario	Endangered Species Act, Statutes of Ontario (2007, c. 6)	Bogbean buckmoth (*Hemileuca* sp.) False-foxglove sun moth (*Pyrrhia aurantiago*) Gypsy cuckoo bumble bee (*Bombus bohemicus*) Mottled duskywing (*Erynnis martialis*) Rusty-patched bumble bee (*Bombus affinis*) Suckley’s cuckoo bumble bee (*Bombus suckleyi*) Northern oak hairstreak (*Satyrium favonius ontario*) Reversed haploa moth (*Haploa reversa*) American bumble bee (*Bombus pensylvanicus*) Dukes’ skipper (*Euphyes dukesi*) Monarch (*Danaus plexippus*) Red-tailed leafhopper (*Aflexia rubranura*) West Virginia white (*Pieris virginiensis*) Yellow-banded bumble bee (*Bombus terricola*)
Quebec	Act Respecting Threatened or Vulnerable Species, Revised Statutes of Quebec (1989, c. E-12.01)	Rusty-patched bumble bee (*Bombus affinis*) Salt marsh copper (*Lycaena dospassosi*) Maritime ringlet (*Coenonympha nipisiquit*)
New Brunswick	Endangered Species Act, Revised Statutes of New Brunswick (2012, c. 6)	Bumble bee, Bohemian cuckoo (*Bombus bohemicus*) Bumble bee, rusty-patched (*Bombus affinis*) Bumble bee, Suckley’s cuckoo (*Bombus suckleyi*) Monarch (*Danaus plexippus*) Ringlet, maritime (*Coenonympha nipisiquit*)
Nova Scotia	Endangered Species Act, Statutes of Nova Scotia (1998, c. 11. s. 1)	Gypsy cuckoo bumble bee (*Bombus bohemicus*) Macropis buckoo bee (*Epeoloides pilosulus*) Monarch (*Danaus plexippus*) Sable Island sweat bee (*Lasioglossum sablense*) Yellow-banded bumble bee (*Bombus terricola*)
Prince Edward Island	None	
Newfoundland	Endangered Species Act, Statutes of Newfoundland and Labrador (2001, c. E-10.1)	Gypsy cuckoo bumble bee (*Bombus bohemicus*) Suckley’s cuckoo bumble bee (*Bombus suckleyi*) Yellow-banded bumble bee (*Bombus terricola*)
Yukon	None	
Northwest Territories	Species at Risk (NWT) Act, Statutes of Northwest Territories (2009, c. 16)	No pollinators listed ^1^
Nunavut	None	

^1^ NWT Species at Risk Committee (SARC) considers gypsy cuckoo bumble bee and McKay’s bumble bee to be data deficient and yellow-banded bumble bee to be not at risk; Suckley’s cuckoo bumble bee is not assessed. SARC assessments enabled by Species at Risk (NWT) Act.

**Table 3:** Requirements within provincial legislation pertaining to beekeeping that potentially reduce risk to native pollinators. Requirements are organized first by general functions, and then by actions (identified in italics). See Table S1 in the Supplementary Materials for example legislative wording for each action identified here.

1. Requirements that track the number and location of honey bee hives.	
	*Registering hives*: Bee Regulation (Alberta Regulation 194/2003; 221/2004, s.3)
	*Identification of hives*: New Brunswick Regulation 2023-13, s.18(1)
	*Registering specific location of hives*: Bee Industry Regulations (Nova Scotia Regulation 319/2007, s.5)
	*Mandatory reporting of movement of honey bee hives/colonies*: Bee Health Regulations (Pursuant to Section 4 of the Animal Health and Protection Act, Revised Statutes of Prince Edward Island. 1988, Cap. A-11.1, s.6(1), s.6(2))
2. Requirements that control the spread of pathogens	
	*Mandatory inspection of bees prior to importation*: Animal Health Regulations (Newfoundland and Labrador Regulation 33/2012, s.7(1), s.7(2))
	*Mandatory quarantine period*: Animal Health Regulations (Newfoundland and Labrador Regulation 33/2012, s.7(3))
	*Mandatory inspection of used honey bee equipment prior to importation*: Bee Industry Regulations (Nova Scotia Regulation 319/2007, s.6(1), s.6(2a), s.6(2b))
	*Mandatory sanitization of equipment prior to importation*: 2024 Nova Scotia Honey Bee Health Importation Protocol
	*Mandatory reporting of disease and pests*: Bees Act (Revised Statutes of Ontario, 1990, c. B.6, s.10)
	*Listing of specific pathogens*: refer to Table 4 for a list by province
	*Restrictions on the source of honey bees and honey bee equipment*: The Apiaries Regulation (Saskatchewan 2005 cA-22.01 Regulation 1, s.4)
3. Requirements that can control competition with native pollinators	
	*Regulations controlling apiaries on public land:* British Columbia Land Use Policy Permission, 2023. Appendix 4. Conditions for temporary apiaries (issued under authority of Lands Act (C.245, Revised Statutes of British Columbia, 1996, s.60))
	*Restricting number of hives*: The City of Dawson Bylaw #12-28, s.6.08
4. Requirements that reduce lethal and sub-lethal effects of pesticides on native pollinators ^1^	
	*Restricting pesticides while flowers are in bloom*: Animal Health Protection Act (Revised Statutes of Quebec, 1964 Ch. P-42, s. 11.12)
5. Requirements that make regulations applicable to domesticated bees in addition to honey bees	
	*Defining imported bee species* (e.g., *honey bees, bumble bees, alfalfa leaf-cutter bees, blue orchard bees*): The Bee Act (Continuing Consolidation of the Statutes of Manitoba c. B15, s.1)

^1^ Although we note this as an existing legislative requirement in some jurisdictions, we do not discuss this topic further because of its complexity. Substantial research has been performed, often on species other than bees, leading to diverse governance outcomes beyond the scope of this paper.

**Table 4:** Pathogens identified in provincial and territorial statutes and regulations: X—identified only; R—reportable; N—notifiable. “Reportable” and “Notifiable” are defined in the text. Two-letter codes for jurisdictions are as follows: YT—Yukon Territory; BC—British Columbia; AB—Alberta; SK—Saskatchewan; MB—Manitoba; ON—Ontario; QC—Québec; NB—New Brunswick; NS—Nova Scotia; PEI—Prince Edward Island; NL—Newfoundland and Labrador.

Common Name	Scientific Name	YT	BC	AB	SK	MB	ON	QC	NB	NS	PEI	NL
Mites												
Asian bee mite sp. 1	*Tropilaelaps mercedesae*			X		X	R	R	R	R		
Asian bee mite sp. 2	*Tropilaelaps clareae*		R	X	X	X	R	R	R	R		
*Euvarroa* genus	*Euvarroa*					X	R		R			
Tracheal mites	*Acarapis woodi*		N	X	X	R	R		X	R	R	R
*Varroa* mites	*Varroa destructor*		N	X	X	X	R	X	R ^1^	R ^1^	R	R
Bees, Wasps, and Hornets												
Africanized honey bee	*Apis mellifera scutellata* and hybrids		R	X	X	X	R	R	R	R	X	
Asian honey bee	*Apis cerana* and species complex		R	X		X	R		R	R	X	
Cape honey bee	*Apis mellifera capensis*		R	X		X	R		R	R	X	
Dwarf honey bee	*Apis florea* and species complex					X	R		R			
Giant honey bee	*Apis dorsata*			X		X	R		R			
Asian predatory wasp	*Apis vilutina*		R									
Northern giant hornet	*Vespa mandarinia*		R	X		X	R		R	R		
Southern giant hornet	*Vespa soror*			X		X			R			
Yellow-legged hornet	*Vespa velutina*			X		X			R		X	
Beetles												
Large African hive beetle sp. 1	*Oplostomus fuligineus*					X	R		R			
Large African hive beetle sp. 2	*Oplostomus haroldi*					X	R		R			
Small hive beetle	*Aethina tumida*	R	R	X	X	R	R	R	X	R	R	X
Moths												
Greater wax moth	*Galleria mellonella*		N			X			X			R
Lesser wax moth	*Achoia grisella*		N			X			X			
Other insects												
Bee louse	*Braula coeca*					X			X			
Fungi												
Chalkbrood	*Ascosphaera apis*		N	X		X			X	X		
*Nosema* sp. 1	*Nosema apis* (*Vairimorpha apis*)		N	X		R	R		X	X		R
*Nosema* sp. 2	*Nosema ceranae* (*Vairimorpha ceranae*)		N	X		X	R		X	R		
Bacteria												
American foulbrood	*Paenibacillus* larvae	R	R	X	N	R	R	R	R	R	R	R
European foulbrood	*Melissococcus plutonius* and bacteria		N	X		R	R		R	R		R
Viruses												
Acute bee paralysis virus			N			X						
Black queen cell virus			N			X						
Chronic bee paralysis virus						X						
Deformed wing virus			N			X						
Israel acute paralysis virus			N			X						
Kashmir bee virus			N			X						
Sacbrood virus	Family Iflaviridae		N	X		X			X	X		
Varroa destructor virus			N			X						

^1^ Reportable for miticide-resistant types only.

## Data Availability

No new data were created.

## Supplementary Materials

The following supporting information can be downloaded from https://www.mdpi.com/article/10.3390/biology14030282/s1: Table S1. Requirements within provincial legislation pertaining to beekeeping that potentially reduce risk to native pollinators. Requirements are organized first by general functions, and then by actions (identified in italics). Each action is followed by example wording directly quoted from existing legislation. Table S2. Sample list of bylaws pertaining to urban beekeeping.

## Abbreviations

The following abbreviations are used in this manuscript:

CFIA	Canadian Food Inspection Agency
COSEWIC	Committee on the Status of Endangered Wildlife in Canada
NWT	Northwest Territories
OIC	Order in Council
SARA	Species at Risk Act
SARC	Species at Risk Committee
