# Supplementary material for: Reducing Risks to Native Pollinators by Introduced Bees: A Review of Canada’s Legislation with Recommendations for Yukon Territory
Source: Biology (Basel). 2025 Mar 11;14(3):282. doi: 10.3390/biology14030282 (PMC11939998; doi:10.3390/biology14030282)
Supplement: Supplementary file 1 [file biology-14-00282-s001.zip › biology-3490638-supplementary.pdf]

Reducing risks to native pollinators by introduced bees: a review of Canada's legislation with recommendations for Yukon Territory.

Leung, M.C.-Y. and Reid, D.G.

The following Tables are referenced in the paper and provide added evidence for some of the points made, and as example wording for anyone interested in developing new legislation.

Table S1. Requirements within provincial legislation pertaining to beekeeping that potentially reduce risk to native pollinators. Requirements are organized first by general functions, and then by actions (identified in italics). Each action is followed by example wording directly quoted from existing legislation.

---

1. Requirements that track the number and location of hives.

Registering hives

Bee Regulation (Alberta Regulation 194/2003 s3;221/2004)

Registration

3 (1) A beekeeper must apply for registration with the Provincial Apiculturist each year.

(2) An applicant for registration must provide the following information to the Provincial Apiculturist:

(a) the full name, address and telephone number of the applicant;

(b) the number of colonies of bees owned by the applicant;

(c) the number of apiaries owned by the applicant and the names of the municipalities in which they are located;

(d) if the applicant has purchased live bees within the preceding 12 months or since the last registration, the names of the persons who provided the bees.

Identification of hives

New Brunswick Regulation 2023-13 s18

Identification of hives

18 (1) For the purpose of subsection 37(1) of the Act, hives shall be identified with the code assigned to the beekeeper on their certificate of registration in numbers and letters at least 2.5 cm high, displayed as follows in a manner that is clearly visible:

(a) if hives are on a pallet, on one hive per pallet;

(b) if hives are not on a pallet, on each hive.

Registering specific location of hives

Bee Industry Regulations (Nova Scotia Regulation 319/2007, s.5)

Information on location of apiaries

5. No later than 24 hours after being requested by the Apiculturist or an inspector, a beekeeper must provide all of the following location information to the Apiculturist or inspector:

(a) the number of hives at each apiary they keep;

(b) the civic address and geographic location of each apiary they keep;

(c) any additional information requested, in enough detail to assist the Apiculturist or inspector in finding the apiary, including specific hives on the beekeeper's land.

Mandatory reporting of movement of honey bees

Bee Health Regulations (Pursuant to section 4 of the Animal Health and Protection Act R.S.P.E.I. 1988, Cap. A-11.1 s.6(1), s.6(2))

6. (1) No person shall transport honey bees into the province unless the person holds a bee health certificate respecting those honey bees, issued pursuant to subsection (2).

(2) The provincial apiarist shall, on application, issue to an applicant a bee health certificate respecting honey bees the applicant wishes to transport into the province, if the applicant provides to the provincial apiarist,

(a) where the honey bees are being imported into the province from outside of Canada, a copy of an import certificate respecting the honey bees, issued by the Canadian Food Inspection Agency pursuant to the Health of Animals Act (Canada); or

(b) where the honey bees are being imported into the province from within Canada,

(i) a certificate, signed and dated by an authorized inspector from the province of origin, not more than 60 days prior to the proposed date of entry, certifying that at least 10% of the honey bees being imported were inspected for infestation by small hive beetles and small hive beetles were not detected during the inspection, and

(ii) a declaration, in the form required by the provincial apiarist, signed and dated by the exporting beekeeper, not more than 60 days prior to the proposed date of entry, confirming that the honey bees being imported were treated, within 300 days preceding the proposed date of entry, with a treatment approved by the provincial apiarist, to reduce or eliminate infestation by honey bee tracheal mites.

2. Requirements that control the spread of pathogens

Mandatory inspection of bees prior to importation

Animal Health Regulations (Newfoundland and Labrador Regulation 33/2012 s.7(1), s.7(2))

7.(1) A person shall not bring honeybees (*Apis mellifera*) or honeybee hives into the province unless accompanied by a certificate signed by an official veterinarian or licensed veterinarian in the place of origin of the honeybees or hives or by an accredited veterinarian or other person approved by the Chief Veterinary Officer certifying that:

(a) the honeybees and hives are free from the following pests:

(i) honeybee tracheal mite (*Acarapis woodi*),

(ii) varroa mite (*Varroa destructor*),

(iii) greater wax moth (*Galleria mellonella*), and

(iv) small hive beetle (*Aethina tumida*);

(b) the apiary from which the honeybees or hives originated was completely free from the pests described in paragraph (a) when the honeybees or hives were taken from the apiary and in the 12 months immediately preceding their departure; and

(c) the honeybees or hives were tested for the pests described in paragraph (a) with negative test results within the 30 days, or such other time as the Chief Veterinary Officer may in his or her discretion prescribe in a particular case, preceding entry to the province.

(2) A person who imports honeybees or honeybee hives shall ensure that honeybees and hives are not exposed to other honeybees or hives during shipment.

Mandatory quarantine period

Animal Health Regulations (Newfoundland and Labrador Regulation 33/2012, s.7(3))

7.(3) Upon entry into the province all honeybees and honeybee hives, regardless of compliance with subsection (1), shall be quarantined on the premises of the importer for a period of 12 months from the date of entry.

Mandatory inspection of used honey bee equipment prior to importation

Bee Industry Regulations (Nova Scotia Regulation 319/2007, s.6(1), s.6(2a), s6(2b))

Import permits

6 (1) In this Section and in Section 4 of the Act, a reference to bees includes the sperm and eggs of bees.

(2) Subject to subsection (3), an import permit may be issued by the Minister only if all of the following conditions are met:

(a) the applicant is a registered beekeeper;

(b) for bees or beekeeping equipment that are being imported from outside of Canada, the beekeeper provides the Apiculturist with a copy of the import permit or permit number issued by Agriculture and Agri-Food Canada that permits the bees or beekeeping equipment to be imported and the Apiculturist is satisfied that

(i) the bees or beekeeping equipment have been sampled in accordance with testing procedures that are acceptable to the Apiculturist, and

(ii) the bees or beekeeping equipment meet the specifications set out in the *Bee Health Importation Protocol* published by the Department of Agriculture;

Mandatory sanitization of equipment prior to importation

2024 Nova Scotia Honeybee Health Importation Protocol

The Bee Import Protocol is established under the *Bee Industry Regulations* made under Section 17 of the *Bee Industry Act* and sets out the conditions in which bees can be imported into Nova Scotia from outside Canada or from another province or territory.

A permit to import used hiveware such as: supers, brood chambers, brood comb, frames, feeders, covers and bottoms, from other Canadian Provinces into Nova Scotia that has been previously used or exposed to honeybees may be issued where risk of introduction of reportable pests and pathogens is considered negligible, and the following criteria are met.

- Hiveware must be treated in an insect-tight, climate-controlled reefer truck for 48h at -12°C, and treatment confirmed by temperature data; or
- Hiveware must be sterilized by a method prescribed by the Provincial Apiculturist such as gamma or electron beam irradiation, chemical disinfection, fumigation, etc. and validated by invoice, certificate or written testimonial of 3rd party sterilization provider or reliable witness.
- Equipment must be inspected by an inspector before use.

Mandatory reporting of disease and pests
Bees Act (Revised Statutes of Ontario, 1990, c. B.6, s.10)
Duty of beekeeper to report disease
10. Every beekeeper who finds that any pest is present or disease exists in his, her or its bees or that his, her or its beekeeping equipment is infected, shall immediately report the presence of the pest or the existence of the disease to the Provincial Apiarist.

Listing of specific pathogens -Refer to Table 3 in the paper for list by province.

Restrictions on the source of honey bees and honey bee equipment
The Apiaries Regulation (Saskatchewan 2005 cA-22.01 Regulation 1, s.4)
Import permits
4 The following are required for the purposes of subsection 6(2) of the Act:
(a) the importation must be from a jurisdiction where disease is not known to exist; or
(b) in the minister’s opinion, the importation is unlikely to result in increased risk of disease in Saskatchewan.

3. Requirements that can control competition with native pollinators

Regulations of apiaries on public land
British Columbia Land Use Policy Permission, 2023
(issued under authority of Lands Act (C.245, R.S.B.C., 1996, s.60))
APPENDIX 4. CONDITIONS FOR TEMPORARY APIARIES
1) The apiary does not conflict with existing Authorizations. Land status must be assessed by accessing…
2) The apiary and its management comply with the Animal Health Act and Bee Regulation including registration and signage requirements.
3) The footprint of the apiary site does not exceed $30m^2$.
4) The apiary does not obstruct access to Crown land outside of the apiary footprint.
5) The apiary is located at least 10m from any private property, road, trail, or any area where this Permission does not apply.
6) The apiary is located at least 100m from any other apiary authorized by this permission.
7) The operator does not maintain more than 10 temporary apiaries under this permission.
8) The apiary is not placed on Crown land for more than 120 days per calendar year.
9) Following removal of colonies, all improvements and equipment are removed and the land is restored to a safe, clean, and sanitary condition within one day.
10) Trees are not cut on Crown land without an appropriate cutting authority.
11) Adequate insurance coverage for the activities being undertaken is maintained.

Restricting number of hives
The City of Dawson Bylaw #12-28, s 6.08
6.08 A maximum of two (2) Beehives may be kept on a lot that is smaller than 0.41 ha; a maximum of four (4) Beehives may be kept on lots that are larger than 0.41 ha.

4. Requirements that reduce lethal and sublethal affects of pesticides on native pollinators

Restricting pesticides while flowers are in bloom
Animal Health Protection Act (Revised Statutes of Quebec, 1964 Ch. P-42, s.11.12)
11.12. No person shall spray, by sprinkler or otherwise, or dust, using chemical or biological products that are toxic to bees, a fruit tree or a plant of a species or category designated by regulation while the fruit tree or plant is in bloom.

5. Requirements that make regulations applicable to domesticated bees in addition to honey bees
Defining imported bee species -e.g. honey bees, bumble bees, alfalfa leaf cutter bees, blue orchard bees<br>The Bee Act (Continuing Consolidation of the Statutes of Manitoba c. B15, s.1)<br>Definitions<br>1. In this Act<br>"bee" means the insect<br>(a) *Apis mellifera*, or<br>(b) *Megachile rotundata*; (« abeille »)

Table S2. Sample list of bylaws pertaining to urban beekeeping.

Municipality or Region	Legislation
Abbotsford, BC	Abbotsford Zoning Bylaw, 2014, Amendment Bylaw No. 432
Birdtail Sioux Indian Reserve, MB	Bee Keeping By Law 15-77
Bonnyville, AB	Land Use Bylaw No. 1667
Burnaby, BC	City of Burnaby- Zoning Bylaw Schedule Number 6
Cariboo Regional District, BC	South Cariboo Area Zoning Bylaw No. 3501, 1999
Chilliwack, BC	Urban Beekeeping Bylaw 2019, No. 4680
Dawson City, YT	Animal Control Bylaw
Delta, BC	Delta Zoning Bylaw No. 7600, 2017
Edmonton, AB	Edmonton Animal Licensing and Control Bylaw No. 13145
Grande Prairie, AB	Animal and Responsible Pet Ownership Bylaw C-1226
Kamloops, BC	City of Kamloops Animal Control Bylaw No. 34-11
Maple Ridge, BC	Maple Ridge Hobby Beekeeping Regulation Bylaw No. 6839-2011
North Vancouver, BC	Bylaw 6474: Bee Keeping Bylaw
Mount Pearl, NL	Mount Pearl Development Regulation 2010
New Westminster, BC	Beekeeping Bylaw No. 6648, 2000
Saskatoon, SK	Animal Control Bylaw 7860, s. 21.1; Zoning Bylaw 8770, s. 5.23
Squamish, BC	District of Squamish Animal Control Bylaw
Surrey, BC	Surrey Bee Keeping By-law, 1974, No. 4362
Terrace, BC	City of Terrace Bylaw No. 2159-2019: A bylaw to provide for regulating, licencing and control of animals within the City of Terrace.
Vernon, BC	City of Vernon Animal Regulation and Animal Pound Bylaw #5252
Whitehorse, YT	Zoning Bylaw 2012-20
Winnipeg, MB	Winnipeg Zoning By-Law
